# Exophytic pancreatic lymphoepithelial cyst incidentally detected in a differentiated thyroid cancer patient on whole-body I-131 scan: a case report

**DOI:** 10.1186/s40792-022-01389-7

**Published:** 2022-02-25

**Authors:** Takahiro Murokawa, Takehiro Okabayashi, Kazuyuki Oishi, Kenta Sui, Motoyasu Tabuchi, Jun Iwata

**Affiliations:** 1 Department of Gastroenterological Surgery, Kochi Health Sciences Center, 2125-1 Ike, Kochi-City, Kochi 781-8555 Japan; 2 Department of Diagnostic Pathology, Kochi Health Sciences Center, 2125-1 Ike, Kochi-City, Kochi 781-8555 Japan

**Keywords:** Pancreatic lymphoepithelial cyst, Thyroid cancer, Radioiodine (I-131) whole-body scintigraphy

## Abstract

**Background:**

Radioiodine (I-131) whole-body scintigraphy (WBS) is a useful modality for identifying functionally preserved thyroid tissue and metastases from differentiated thyroid cancer (DTC); however, the specificity of I-131 uptake is limited, and its accumulation in the pancreas has not been well described.

**Case presentation:**

A 70-year-old male patient with DTC who had previously undergone total thyroidectomy (pT3N1bM0 Stage IV) received radioiodine treatment at our facility. After treatment, an I-131 WBS revealed abnormal I-131 uptake in the head of the pancreas. Computed tomography identified a round hypodense mass (10 × 20 mm) adjacent to the pancreas head that was impervious to fluorodeoxyglucose (^18^F-FDG) during subsequent ^18^F-FDG-positron emission tomography. A diagnosis of pancreatic metastasis from the DTC could not be excluded; therefore, local resection was performed for diagnostic certainty and treatment. Histopathology confirmed the mass to be an exophytic lymphoepithelial cyst (LEC) of the pancreas. The patient also had a transient pancreatic leak which spontaneously resolved after surgery, and he was discharged from the hospital on postoperative day 8.

**Conclusion:**

To the best of our knowledge, this is the first reported case of an exophytic pancreatic LEC producing a false-positive result during I-131 WBS. Knowledge of all potential I-131 false-positive findings may help improve the management of patients with DTC and circumvent misdiagnoses.

## Background

Radioiodine (I-131) whole-body scintigraphy (WBS) is routinely used to detect remnants of functioning thyroid tissue, recurrent neoplasia, or distant metastases in patients with differentiated thyroid cancer (DTC) after undergoing postoperative radioiodine therapy [1]. Post-therapeutic I-131 WBS applied to the detection of functioning thyroid tissue is highly sensitive but sometimes non-specific, and the frequent incidence of false-positive findings is a known clinical challenge. The interpretation of I-131 WBS findings is complex, and misdiagnosis may result in unnecessary administration of radioiodine therapeutic doses or implementation of inappropriate surgical procedures. Greater knowledge of the numerous patterns of false-positive findings will help guide effective clinical management of patients with DTC [2]. DTC has an indolent oncological nature and patient outcome is usually excellent after the execution of appropriate therapeutic measures [3–5]. Metastasis is rare but is associated with cancer-related mortality in patients with DTC. Metastases from DTC mainly occur in the lungs, bones, and brain [6], and there are very few reports of their occurrence in the pancreas [7]. Surgical resection has been demonstrated as a beneficial therapeutic option in selected patients with metastatic DTC; otherwise, multi-kinase inhibitors are often palliatively applied [8].

In the present case, a routine I-131 WBS of a 70-year-old patient previously diagnosed and treated for DTC revealed abnormal uptake of radioiodine in the pancreas. A hypodense mass identified near the pancreas head could not be excluded as a pancreatic metastatic neoplasm; however, pathological examination of the tumor tissue after resection confirmed a final diagnosis of pancreatic lymphoepithelial cyst (LEC). The patient recovered well with minimal surgical management. This case has been reported in line with the SCARE criteria [9].

## Case report

**Fig. 1:**
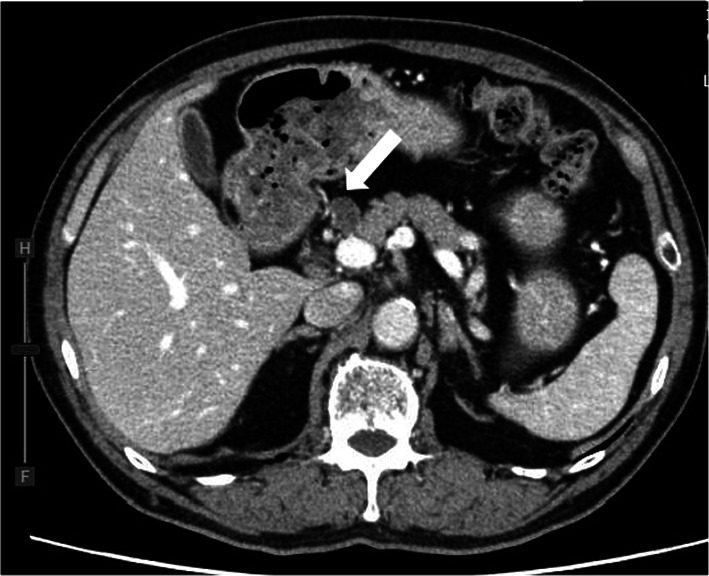
Radioiodine whole-body scintigraphy demonstrated uptake of I-131 in the thyroid bed and the pancreas head (white arrow)

**Fig. 2:**
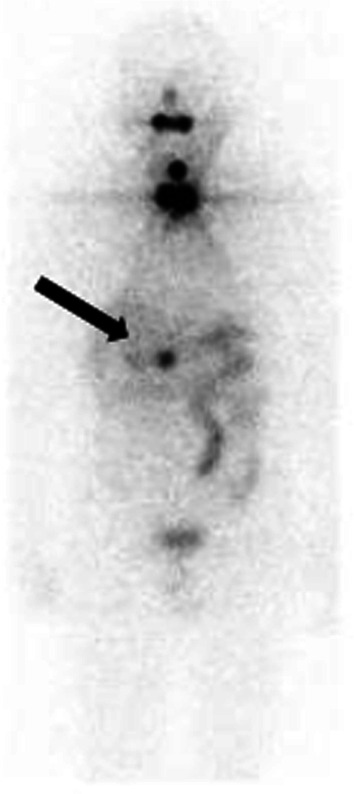
Computed tomography (CT) scan showing a small round hypodense mass (10 × 20 mm in size) adjacent to the head of the pancreas (black arrow)

The patient in this case is a 70-year-old man who had previously undergone total thyroidectomy for papillary thyroid cancer with a pathological staging of pT3N1bM0 (stage IV). Treatment with I-131 (13.7 Gbq) commenced 11 months after initial surgery. After treatment, a routine I-131 WBS showed focal uptake in the thyroid bed and near the head of the pancreas (Fig. 1). Computed tomography (CT) identified a round low dense cystic mass (10 × 20 mm) adjacent to the head of the pancreas (Fig. 2), but no uptake in the mass was observed during fluorodeoxyglucose-positron emission tomography (^18^F-FDG-PET). Retrospectively reviewed, the preoperative CT images before thyroidectomy showed the same lesion with slightly small size (10 × 18 mm). Serum levels of thyroglobulin (Tg) and CA 19-9 were normal (0.59 ng/ml and 14.1 U/ml, respectively). However, open surgical examination was decided to either confirm or exclude the possibility of metastasis from the original DTC.

**Fig. 3:**
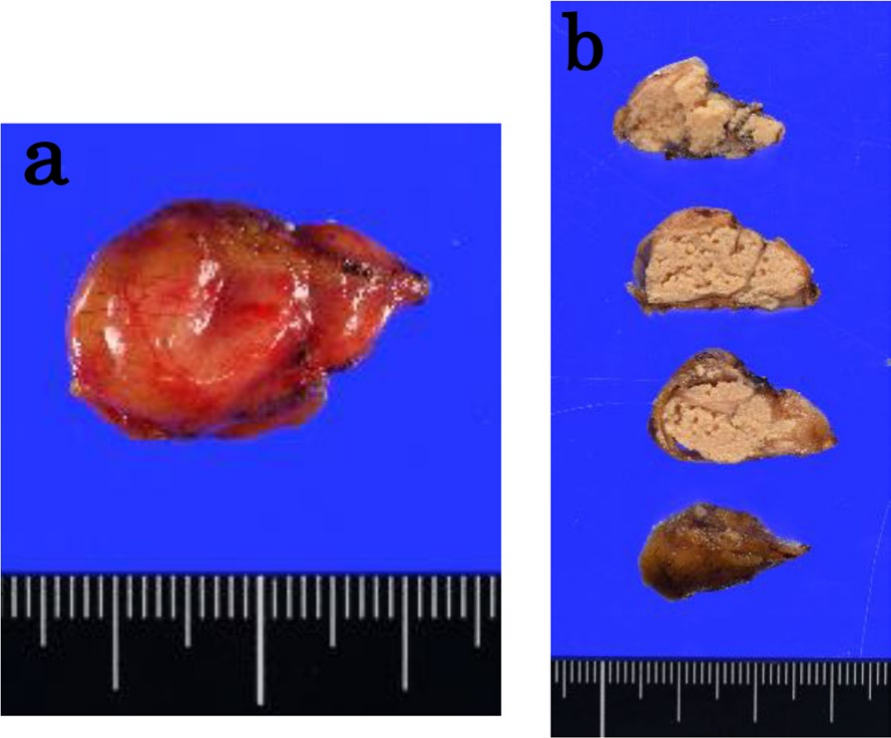
Macroscopic pathological findings. **a** The resected tumor was round and 10 × 20 mm in size. **b** The growth was a multiloculated cyst with a thin capsule containing a yellowish-white granular substance

**Fig. 4:**
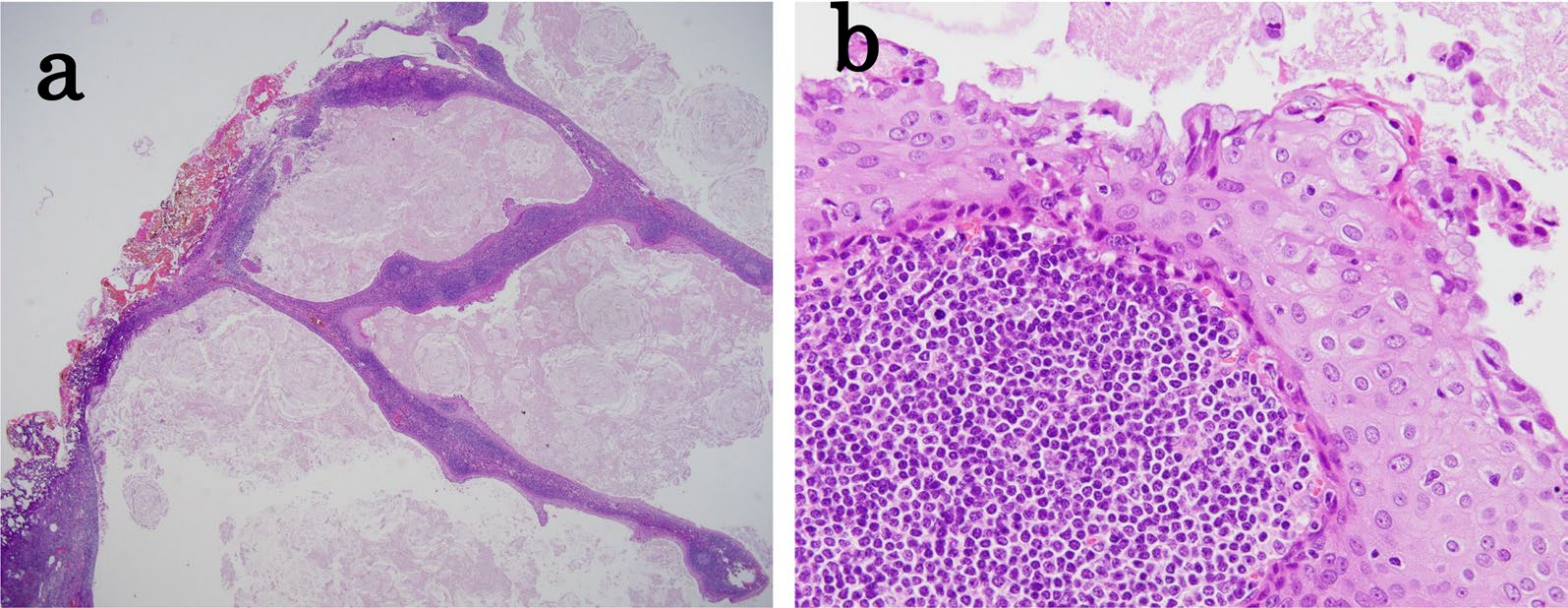
Microscopic pathological findings. **a** The multilocular cyst was segregated by a band of lymphoid tissue (H&E staining; ×40). **b** The cystic wall was lined with stratified squamous epithelium and mucinous cells, and the cystic contents were keratinized (H&E staining; ×400). H&E, hematoxylin and eosin

Intraoperative visual inspection revealed the cyst to be adhered to the pancreatic head. Tumor enucleation was performed, and the tumor was filled with cheesy, granular, and yellowish-white contents (Fig. 3). The tumor was resected, and pathological findings revealed a multiloculate cyst with keratinizing stratified squamous and cuboidal epithelium filled with mucus cells lining the cystic walls. Lymphoid tissue containing lymphoid follicles with germinal centers was found beneath the squamous epithelium (Fig. 4), confirming a final diagnosis of an exophytic LEC of the pancreas. The patient had a transient pancreatic leak, which spontaneously resolved, and he was discharged on postoperative day 8.

## Discussion

Radioiodine WBS is routinely used to screen for local or distant metastases in patients with DTC after having undergone thyroidectomy and subsequent radioiodine therapy [1]. The scan takes advantage of functioning thyroid tissue’s abilities to concentrate, organize, and accumulate radioiodine via the activities of sodium iodide symporters [10]. In this way, I-131 WBS is highly sensitive for thyroid tissue but may lack specificity if I-131 uptake occurs elsewhere, and false-positive findings are common in clinical practice.

There are various possible causes of false-positivity on I-131 WBS after thyroidectomy for DTC: functional sodium iodide symporter expression in normal tissue, benign and malignant tumor, metabolism of radioiodinated hormone, retention of radioiodinated body fluids associated structural change, retention and uptake of radioiodine in inflamed tissue, contamination by physiologic radioiodine uptake [11, 12]. False-positive I-131 WBS findings have been associated with cystic diseases of variable origins, including the nasolacrimal sac, pericardium, bronchial tree, thymus, breast, liver, kidneys, ovaries, epithelium, and skin [13–22]. There is no literature on I-131 avidity in any pancreatic cystic disease, including LECs. Although the mechanism of I-131 uptake in cystic tissue remains unclear, it has been proposed that radioiodine enters cysts through incomplete active transportation pathways or by passive diffusion, and it is then retained because of a relatively slow exchange of water and chemical substances between cysts and surrounding tissues [2]. LECs of the parotid gland are also known to produce false-positives during I-131 WBS [22]; however, to the best of our knowledge, this is the first reported case of false-positivity caused by I-131 uptake in a pancreatic LEC.

The preoperative findings of abnormal I-131 uptake in the pancreas suggested the possible occurrence of metastatic DTC. Patients with DTC are known to experience unusual metastases; however, DTC metastasis to the pancreas is extremely rare, and only 24 cases have ever been reported in the literature [7]. Several clinical observations in this case cast doubt on the differential diagnosis of metastatic neoplasia of the pancreas or intra-abdominal lymph node metastasis from the primary DTC and ectopic thyroid. Firstly, DTC metastases are often broadly disseminated and metastasis to the pancreas is a lengthy process; it is unlikely for a pancreatic metastatic lesion to be detected independently from metastases in other, more common locations like the lung, bones, and liver [7, 8]. Typically, pancreatic metastases no longer exhibit avidity for radioiodine, suggesting suppression of sodium iodide symporter [8, 23]. In addition, loss of I-131 uptake may indicate tumor differentiation and should correlate with tumor staging by ^18^F-FDG-PET [24]. Intra-abdominal lymph node metastasis from DTC is also extremely rare and just one case was reported by Niederle et al. [25]. Relating to lymph node metastasis from DTC, mediastinal lymph node metastasis from DTC is known to show I-131 or ^18^F-FDG-PET avidity [26]. Neither observation corresponded with the preoperative findings in this case; however, the differential diagnosis could not be excluded without intraoperative assessment. Consequently, surgical resection and pathological examination revealed the mass to be an LEC that had mimicked metastatic neoplasms or ectopic thyroid tissue during preoperative assessments.

Ectopic thyroid tissue in the pancreas may be a differential diagnosis based on the I-131 WBS findings. However, such an occurrence is exceedingly rare, and only 3 cases of intra-abdominal ectopic thyroid tissue have ever been reported at sites including the adrenal gland, gall bladder, porta hepatis, mesentery, liver, spleen, retroperitoneum, duodenum, and jejunum. Surgical resection is also commonly applied to ruling out malignancy when this rare occurrence is encountered in clinical practice [27–29]. Overall, recognition of all false-positive I-131 WBS patterns is important for the effective clinical management of patients with DTC.

Pancreatic LECs are relatively rare benign tumors; since first being described by Luchtrach and Schriefers in 1985, over 200 cases of pancreatic LECs appear in the literature, thereby accounting for approximately 0.5% of all pancreatic cysts [30]. LECs mostly occur in middle-aged men and arise in all parts of the pancreas with almost equal incidence. The lesion often protrudes from the pancreas parenchyma and many cases appear to be peripancreatic rather than intrapancreatic [31, 32]. Pathologically, LECs appear as uni- or multilocular lesions in the pancreatic or peripancreatic lymph nodes and consist of keratinizing squamous epithelial cells with lymphoid tissue involvement [33]. On MRI, LECs are mostly hypointense on T1-weighted MR images and hyperintense on T2-weighted images while their contents of keratinized material appear as a hyperintense on T1-weighted MR images [34, 35]. LECs are usually selected for surgical resection because of challenges faced during preoperative diagnosis. Elevation of serum CA 19-9 levels are noted in approximately 50% of pancreatic LEC cases, whereas carcinoembryonic antigen levels are typically within normal range. The non-uniformity of serum findings poses a notable diagnostic challenge. Further, presentations of LECs during preoperative radiology display inter-patient variability and are often difficult to distinguish from other pancreatic cystic lesions, such as serous cystic neoplasms and mucinous cystic neoplasms, intraductal papillary mucinous neoplasms, and dermoid and epidermoid cysts [36]. In terms of etiology, LECs have been hypothesized to develop from benign epithelium or ectopic pancreatic tissues in a peripancreatic lymph node, aberrant positioning of branchial cleft cysts at embryogenesis, or squamous metaplasia in an intrapancreatic duct. The former hypothesis of the development of epithelial remnants in abdominal lymph node is most preferred and could be compatible with this case [32, 37].

Preoperative attempts at cytopathologic assessment usually involve endoscopic ultrasound-guided fine-needle biopsy (EUS-FNA) [38]. The sensitivity and specificity of EUS-FNA is unsatisfactory (accurate diagnosis in approximately 50% of cases); commonly, contamination of the aspirate with mucinous or glandular epithelium from the intestine that may mimic cystic neoplasia further limits diagnostic certainty by this method [39]. Furthermore, there is concern regarding possible dissemination of biopsy material originating from a potentially malignant tumor [40]. However, recent case studies performed by Groot et al. identified key imaging features that help in the preoperative diagnosis of pancreatic LECs, including an exophytic growth pattern, the absence of pancreatic duct dilatation, and the presence of squamous cells, cholesterol crystals, and keratin in material recovered by EUS-FNA [32]. In our case, conservative treatment and serial follow-up may have been appropriate based on the I-131 avidity, normal serum Tg levels, morphological findings, and ^18^F-FDG-PET findings. Considering clinical policy for addressing pancreatic lesions, surgical resection was deemed necessary to rule out all possibilities of malignancy [32, 41]. In some cases, LECs of the pancreas may become enlarged and undergo inflammatory changes; therefore, enucleation or local resection of the tumor was considered the acceptable approach in this case [42, 43]. On the other hand, additional MRI examination also should have been examined and EUS-FNA could help the differential diagnosis.

In conclusion, we present a rare case of pancreatic LEC which produced false-positive radioiodine uptake during routine WBS of a patient previously diagnosed with DTC who had undergone total thyroidectomy and subsequent radioiodine treatment. A better understanding of the false-positivity patterns associated with I-131 WBS is critical for the proper management of patients with DTC.

## Data Availability

All data generated or analyzed during this study are included in the published article.
